# Prevalence and Trends of Handgrip Strength Asymmetry in the United States

**DOI:** 10.20900/agmr20230006

**Published:** 2023-06-25

**Authors:** Ryan McGrath, Justin J. Lang, Brian C. Clark, Peggy M. Cawthon, Kennedy Black, Jacob Kieser, Brooklyn J. Fraser, Grant R. Tomkinson

**Affiliations:** 1 Healthy Aging North Dakota (HAND), North Dakota State University, Fargo, ND 58102, USA; 2 Department of Health, Nutrition, and Exercise Sciences, North Dakota State University, Fargo, ND 58108, USA; 3 Fargo VA Healthcare System, Fargo, ND 58102, USA; 4 Department of Geriatrics, University of North Dakota, Grand Forks, ND 58202, USA; 5 Alliance for Research in Exercise, Nutrition and Activity (ARENA), University of South Australia, Adelaide, 5000, Australia; 6 Centre for Surveillance and Applied Research, Public Health Agency of Canada, Ottawa, ON K1A0K9, Canada; 7 School of Epidemiology and Public Health, University of Ottawa, Ottawa, ON K1N6N5, Canada; 8 Ohio Musculoskeletal and Neurological Institute, Ohio University, Athens, OH 45701, USA; 9 Department of Biomedical Sciences, Ohio University, Athens, OH 45701, USA; 10 Division of Geriatric Medicine, Ohio University, Athens, OH 45701, USA; 11 California Pacific Medical Center Research Institute, San Francisco, CA 94107, USA; 12 Department of Epidemiology and Biostatistics, University of California-San Francisco, San Francisco, CA 94143, USA; 13 Menzies Institute for Medical Research, University of Tasmania, Hobart, 7001, Australia

**Keywords:** aging, epidemiology, hand strength, muscle strength, muscle strength dynamometer, muscle weakness, sarcopenia

## Abstract

**Background:**

Strength asymmetries are a type of muscle function impairment that is associated with several health conditions. However, the prevalence of these asymmetries among adults from the United States remains unknown. We sought to estimate the prevalence and trends of handgrip strength (HGS) asymmetry in American adults.

**Methods:**

The unweighted analytic sample included 23,056 persons aged at least 50-years with information on HGS for both hands from the 2006–2016 waves of the Health and Retirement Study. A handgrip dynamometer measured HGS, with the highest recorded values for each hand used to calculate asymmetry. Persons were categorized into the following asymmetry severity categories: (1) >10%, (2) >20.0%, and (3) >30.0%. Survey weights were used to generate nationally-representative asymmetry estimates.

**Results:**

Overall, there were no statistically significant trends in HGS asymmetry categories over time. The prevalence of HGS asymmetry in the 2014–2016 wave was 53.4% (CI: 52.2–54.4), 26.0% (CI: 25.0–26.9), and 11.7% (CI: 10.9–12.3) for asymmetry at >10%, >20%, and >30%, respectively. HGS asymmetry was generally higher in older Americans compared to middle-aged adults at each wave. In the 2014–2016 wave, >30% asymmetry prevalence was 13.7% (CI: 12.7–14.6) in females and 9.3% (CI: 8.4–10.2) in males. Some differences in asymmetry prevalence by race and ethnicity were observed.

**Conclusions:**

The prevalence of asymmetry was generally high, especially in women and older adults. Ongoing surveillance of strength asymmetry will help monitor trends in muscle dysfunction, guide screening for disablement, identify subpopulations at risk for asymmetry, and inform relevant interventions.

## INTRODUCTION

Handgrip dynamometers are well-utilized tools in clinical and epidemiological settings for collecting handgrip strength (HGS) [1]. Measures of HGS conveniently assess strength capacity and muscle function [2]. Age-related declines in physical function often begin with muscle dysfunction [3]. Deficits in muscle function are observed when weakness is present [3], whereby HGS is below a pre-specified cut-point [4]. Weakness is associated with wide-ranging health conditions such as chronic cardiometabolic diseases, Alzheimer’s disease and related dementias, and functional disability [5]. Therefore, it is not surprising that HGS is considered a critical biomarker of aging that should be included as part of routine geriatric healthcare examinations [6].

Indeed, low HGS is associated with several adverse health outcomes [5]. Protocol guidelines for measuring HGS recommend that persons squeeze a handgrip dynamometer with maximal effort on each hand for multiple trials, and the highest recorded HGS value be included regardless of hand [7]. However, the other non-maximal, but valid HGS measurements of the other hand are generally disregarded [7]. Methods for better utilizing non-maximal HGS measures have emerged. Specifically, examining the differences between the highest recorded HGS values on each hand is used for determining bilateral strength asymmetry. HGS asymmetry can be instantaneously evaluated from HGS protocols that recommend persons squeeze a handgrip dynamometer with maximal effort on each hand [7].

Strength asymmetries, as determined with HGS asymmetry, are associated with neurodegenerative disorders [8], falls [9], and early all-cause mortality [10]. Moreover, HGS asymmetry, as another type of muscle function impairment, may occur before weakness [11], and the presence of weakness and asymmetry together may signify more severe muscle dysfunction [12]. While no well-validated and utilized HGS asymmetry cut-points exist, asymmetry could be considered a muscle function impairment when strength between hands is >10%, >20%, or >30%. Problems with generalizing HGS as an overall marker of muscle function also exist [13]. Muscle function is comprised of several characteristics apart from maximal strength [14], such as muscle fatigability and coordination, and these broader muscle function characteristics are not assayed by HGS alone [7].

Several studies have examined the prevalence of weakness in Americans [15–17]. Given that HGS asymmetry is another type of muscle function impairment that can be evaluated alongside weakness, the prevalence of HGS asymmetry should likewise be examined, but such information remains absent. Providing the prevalence of HGS asymmetry for Americans will allow for comparisons to other forms of muscle dysfunction such as weakness, inform primary, secondary, and tertiary interventions for disablement, and identify at-risk sub-populations wherein strength asymmetry is elevated. Accordingly, we sought to determine the prevalence and trends of HGS asymmetry in American adults.

## MATERIALS AND METHODS

### Participants

Our unweighted initial sample included 23,089 participants aged at least 50-years from the 2006–2016 waves of the Health and Retirement Study (HRS) with HGS measured on both hands. The HRS utilizes a longitudinal-panel design to examine health and economic factors during aging [18]. New cohorts of participants are added to the HRS every 6-years [19]. Participants engage in core interviews biennially and are followed until death [18]. Sampling weights are provided by the HRS to generate nationally-representative data [19]. More details about the HRS are available elsewhere [20].

Beginning in the 2006 wave, the HRS conducted enhanced face-to-face interviews in participant residents to collect physical measures such as HGS [18]. These enhanced interviews occurred at alternating waves with half of the full sample randomly selected to participate. Accordingly, we combined the 2006–2008, 2010–2012, and 2014–2016 waves so that the random half samples that completed the HGS testing could be evaluated under a uniform time-period. Written informed consent was provided by all HRS participants and the University’s Health/Behavioral Sciences Committee Institutional Review Board approved the HRS protocols (HUM00061128; 09/20/1990–12/31/23).

### Measures

A Smedley spring-type handgrip dynamometer (Scandidact; Odder, Denmark) was used to measure HGS. Persons reporting surgery, swelling, inflammation, severe pain, or an injury in their hands during the previous 6-months did not perform HGS testing. A handgrip dynamometer was fitted to each participant’s hand size and trained interviewers provided HGS test instructions. A practice trial was permitted with the dominant hand. Starting on the non-dominant hand, participants squeezed the dynamometer with maximal effort while standing with their arm at the side elbow at 90°. If a participant had difficulty grasping the dynamometer they were allowed to complete HGS testing with their arm on a supporting surface. Further, those unable to stand while engaging in HGS testing could complete the testing in a seated position. Participants completed two trials on each hand, alternating between hands. More details about the HGS testing procedures used in the HRS are available elsewhere [21].

The highest recorded HGS values from each hand were used in the calculation of HGS asymmetry ratio: *(strongest HGS (kilograms)/strongest HGS on the other hand (kilograms))* [22]. Therefore, all asymmetry ratios were ≥1.0. Given that wider differences in strength between hands suggests severer asymmetries, participants were classified into the following HGS asymmetry groups: (1) >10%, (2) >20%, and (3) >30% [22–26].

Age, sex (male, female), race and ethnicity (Hispanic, non-Hispanic black, non-Hispanic white, non-Hispanic other (included American Indian, Alaskan Native, Asian, Native Hawaiian, Pacific Islander)) were self-reported. Persons aged 50–64 years were considered middle-aged, whereas those ≥65-years were older adults. Participants with missing sociodemographic information were excluded (*n* = 33).

### Statistical Analysis

All analyses were conducted with SAS 9.4 software (SAS Institute; Cary, NC, USA). HRS analytic recommendations were used for guiding analyses to account for the complex sampling design and generating nationally-representative prevalence estimates. Asymmetry prevalence estimates were presented as a weighed percentage and 95% confidence interval (CI) for each asymmetry category. The asymmetry prevalence estimates were shown as overall, and stratified by age group, sex, and race and ethnicity for the merged HRS waves (i.e., 2006–2008, 2010–2012, 2014–2016). These asymmetry estimates were considered our principal findings.

Individual multilevel logistic regression models for examining trends in the HGS asymmetry categories were performed for overall, age group, sex, and race and ethnicity. To account for the longitudinal design, repeated measures of individual participants in multiple waves were modelled using a random intercept for each participant. In each model, the dichotomous outcome was the specific merged HGS asymmetry categories (i.e., >10%, >20%, >30%). For the overall model, the only predictor was time (e.g., wave). To examine trends by age group, the model was adjusted for time, age group (reference: middle-aged), and the interaction of time and age group. Likewise, the sex-specific model adjusted for time, sex (reference: female), and time-by-sex interaction. The model assessing race and ethnicity included a predictor for time, race and ethnicity (reference group: non-Hispanic White), and the interaction between such effects. These models included survey weights and an alpha level of 0.05 was utilized.

A Sankey Bar Chart was produced to depict the fluidity in the prevalence of Americans with differing levels of HGS asymmetry. In short, Sankey Bar Charts allow for changes within categorical groups to be observed over time [27]. As a supplementary analysis, the same analytical framework from our principal analyses was used for determining asymmetry prevalence with separated HGS asymmetry categories: (1) 0.0–10.0%, (2) 10.1%–20.0%, (3) 20.1%–30.0%, and (4) >30%. The prevalence estimates for these separated HGS asymmetry categories were again presented as overall and stratified by age group, sex, and race and ethnicity. Further, we estimated the prevalence of HGS asymmetry within different older adult age groups (young old: 65–74 years; middle old: 75–84 years; old-old: ≥85 years). Given that these analyses were additional, the findings from these analyses were included as a supplementary and minimally discussed.

## RESULTS

Table 1 presents the unweighted descriptive characteristics of the 23,056 participants. Overall, persons were aged 64.4 ± 10.5 years and were mostly female (57%). Table 2 shows the overall prevalence of HGS asymmetry. In the 2006–2008 waves, there were more Americans with >10% asymmetry (52.3%; CI: 51.2–53.3) relative to persons with >20.0% asymmetry (24.7%; CI: 23.8–25.5) and >30% asymmetry (11.3%; CI: 10.7–11.9). Similar findings were observed in the 2010–2012 and 2014–2016 waves. There were no significant trends in HGS asymmetry categories over time.

The prevalence of HGS asymmetry by age group is in Table 3. Some differences for the prevalence of Americans in HGS asymmetry category existed between age groups. For example, the prevalence of older Americans with >30% asymmetry was 13.1% (CI: 12.2–13.8) in the 2006–2008 waves, 13.3% (CI: 12.4–14.1) in the 2010–2012 waves, and 13.4% (CI: 12.4–14.2) in the 2014–2016 waves. However, these prevalence estimates for >30.0% asymmetry in middle-aged Americans were lower relative to those for older Americans at each time-period: 9.6% (CI: 8.6–10.5) for the 2006–2008 waves, 9.1% (CI: 8.3–9.9) for the 2010–2012 waves, and 10.1% (CI: 9.1–11.0) for the 2014–2016 waves.

Table 4 presents the prevalence of HGS asymmetry by sex. Differences in the prevalence of asymmetry existed between males and females, such that males had a higher estimated prevalence of HGS asymmetry at >10% (*p* < 0.001), >20% (*p* < 0.001), and >30% (*p* < 0.001). For example, the prevalence of >30% asymmetry in males was 8.9% (CI: 8.0–9.7), 9.2% (CI: 8.3–10.0), and 9.3 (CI: 8.4–10.2) for the 2006–2008, 2010–2012, and 2014–2016 waves, respectively. Compared to males, the prevalence of >30% asymmetry at each time-period was higher in females: 13.3% (CI: 12.3–14.1) for the 2006–2008 waves, 12.4% (CI: 11.5–13.2) for the 2010–2012 waves, and 13.7% (CI: 12.7–14.6) for the 2014–2016 waves.

The prevalence of HGS asymmetry by race and ethnicity is shown in Table 5. Prevalence estimates for asymmetry differed between races and ethnicities. For example, the prevalence of >30.0% asymmetry in non-Hispanic blacks was 13.7% (CI: 11.8–15.6) in the 2006–2008 waves, 13.3% (CI: 11.6–14.8) in the 2010–2012 waves, and 15.4% (CI: 13.5–17.3) in the 2014–2016 waves. These prevalence estimates observed for severe asymmetry in non-Hispanic blacks were higher than what was observed in non-Hispanic whites, such that the prevalence of >30.0% asymmetry was 10.7% (CI: 10.0–11.4) in the 2006–2008 waves, 10.0% (CI: 9.7–11.0) in the 2010–2012 waves, and 10.8 (CI: 10.0–11.6) in the 2014–2016 waves.

**Figure 1.**
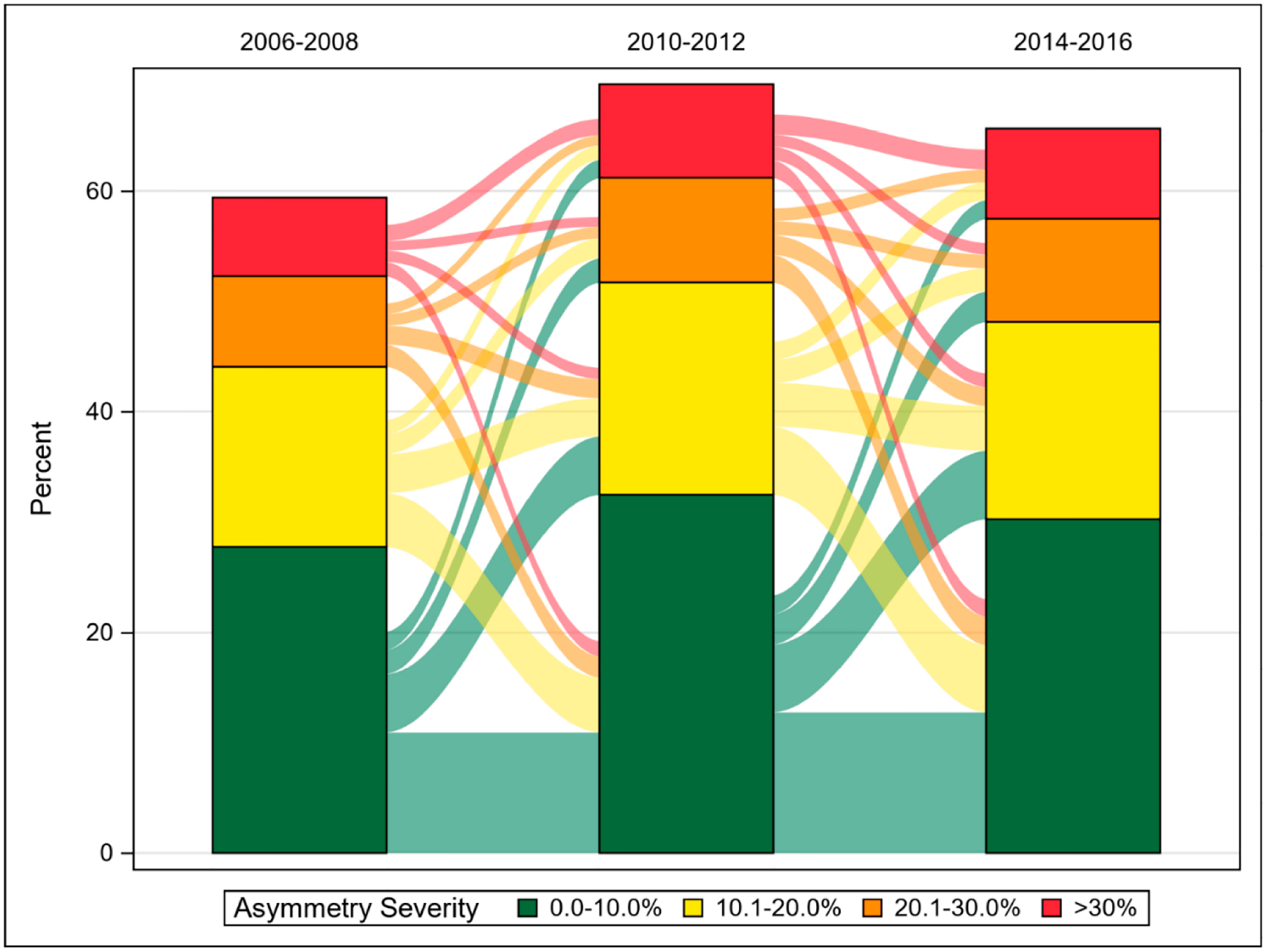
Sankey Bar Chart for Handgrip Strength Asymmetry.

Figure 1 shows a Sankey Bar Chart that displays the fluidity in HGS asymmetry for each severity category over the study period. Table 6 shows the results for the HGS asymmetry trends analyses. Supplementary File 1 presents the results for the overall prevalence of the separated HGS asymmetry categories. Estimated asymmetry prevalence remained relatively consistent within each cut-point across waves. For example, HGS asymmetry >30.0% was 11.3% (CI: 10.7–11.9) in the 2006–2008 waves, 10.9% (CI: 10.3–11.5) in the 2010–2012 waves, and 11.7% (CI: 10.9–12.3) in the 2014–2016 waves. The prevalence of the individualized asymmetry categories by age group, sex, and race and ethnicity are presented in Supplementary Files 2, 3, and 4 respectively. The estimated asymmetry prevalence at 10.1%–20.0% was 27.8% (CI: 26.3–29.2) for middle-aged adults in the 2006–2008 waves, but alternatively was 27.5% (CI: 26.4–28.4) for older adults in the 2006–2008 waves. Supplementary File 5 also shows the prevalence of HGS asymmetry in persons categorized as young old, middle old, and old-old.

## DISCUSSION

The principal findings of this investigation revealed that HGS asymmetry is prevalent among adults in the United States. Specifically, over half of Americans aged at least 50-years were living with asymmetry >10% at each wave, but there were fewer Americans with asymmetry >20.0% or >30.0%. More older Americans have HGS asymmetry, especially at >30.0% asymmetry, compared to middle-aged Americans. The prevalence of HGS asymmetry was generally higher in females relative to males. Some differences emerged when examining HGS asymmetry by race and ethnicity. The presence of asymmetric HGS is fluid such that asymmetry status may worsen, remain consistent, or improve over time. Given that HGS asymmetry is linked to adverse health outcomes, but is prevalent in American adults, consistent screening for asymmetry alongside weakness is recommended especially because modifications to current HGS protocol guidelines are not needed.

Our findings are consistent with other studies that have reported on the prevalence of weakness among American adults. For example, multiple cut-points exist for determining weakness, which in turn, influences how the prevalence of weakness is estimated in Americans [17]. Batsis et al. [15]. also utilized different weakness cut-points for observing the relationship between weakness prevalence and functional limitations, wherein the choice of cut-points similarly influenced the findings. We were consistent in this regard. By applying three HGS asymmetry cut-points, we found that prevalence estimates declined with increased HGS asymmetry severity. While the asymmetry cut-points used in other investigations informed our categorization of HGS asymmetry, developing a more established asymmetry cut-point will help to create consistency in HGS asymmetry definitions [22–26].

The prevalence of asymmetry is different among those with specific sociodemographic characteristics. For example, the prevalence of asymmetry was especially high in older Americans. Several reasons exist that could explain age-related increases in HGS asymmetry. Peripheral factors such as pain (e.g., osteoarthritis) resulting in inhibitory feedback to suppress force output on a given hand but not the other. The greater prevalence of strength asymmetry in older Americans could be attributed to central neural factors that range from more accelerated aging of the right cerebral hemisphere [28,29], to age-related reductions in the corpus callosum connectively between homotopic regions of the left and right cerebral hemispheres [30]. More work is required to better understand the neuromuscular mechanisms underlying HGS asymmetry.

Our findings likewise revealed that the prevalence of asymmetric HGS was generally higher in females. Previous work has shown that evaluating HGS asymmetry for adverse health conditions could be especially important for females [31]. The prevalence of HGS asymmetry by race and ethnicity in our study is consistent with other work examining weakness prevalence by race and ethnicity [17]. Continuing to monitor HGS asymmetry by distinct sociodemographic characteristics help to inform interventions targeting at risk populations.

Although weakness, using absolute or body size normalized cut-points, is a long-standing marker of muscle dysfunction, asymmetry may also provide an additional measure of muscle dysfunction that may occur before weakness is observed, and both weakness and asymmetry could represent a more severe muscle function impairment than weakness or asymmetry alone [11]. Asymmetry assessments can likewise be immediately adopted in recommended HGS protocols [7]. Accordingly, ongoing surveillance of both asymmetry and weakness may help to identify groups at greater risk for muscle dysfunction and inform germane interventions. Implications from such work may help to determine how weakness and asymmetry can specify prognosis, and fluidity may associate with age-related, clinically-relevant health outcomes.

Some limitations should be acknowledged. Certain races and ethnicities such as non-Hispanic Asian are not specified in the HRS. The enhanced face-to-face interviews in the HRS occur on alternating waves, thereby explaining why we combined alternative waves for our analyses. Although unavailable, established asymmetry cut-points may have guided our analyses, but we nonetheless examined HGS asymmetry with multiple cut-points. Exclusions for morbidities and disabilities that may influence HGS asymmetries were not performed because such exclusions fall outside the scope of our investigation. A well-established universal cut-point for asymmetric HGS remains absent, yet needed, but our investigation utilized cut-points that were informed from previous work. We used a ratio for determining asymmetry, but other methods of defining asymmetry such as absolute percent difference also exist. The HRS provides nationally-representative information for Americans aged at least 50-years, but other nationally-representative data sources that include HGS measurements on both hands exist and should be utilized to examine the prevalence of asymmetries in sociodemographic groups not evaluated herein.

## CONCLUSIONS

This investigation revealed that many Americans aged at least 50-years have asymmetric HGS. The prevalence of asymmetry was especially high for older adults and females. Asymmetry status was relatively fluid, thereby suggesting that opportunities to correct asymmetries through referrals are possible. We recommend asymmetry be examined alongside maximal HGS and the implementation of asymmetry in current protocols that collect HGS on both hands should be instantaneous. Surveillance of asymmetry status will help in monitoring the significance of asymmetry in the United States and may inform interventions seeking to prevent and treat muscle dysfunction. Reducing bilateral strength asymmetries may help the quickly growing older American demographic retain their independence and preserve quality of life.

## Supplementary Material

Supplementary material

## Figures and Tables

**Table 1.** Unweighted Descriptive Characteristics of the Participants.

Variables	Total Overall Sample (*n* = 23,056)	2006–2008 (*n* = 13,698)	2010–2012 (*n* = 16,051)	2014–2016 (*n* = 15,130)
Age (years)	64.4 ± 10.5	68.5 ± 9.9	66.3 ± 10.7	66.9 ± 10.6
Age Group (*n* (%))				
Middle-Aged (*n* (%))	12,726 (55.2)	4927 (36.0)	7922 (49.4)	7303 (48.3)
Older (*n* (%))	10,330 (44.8)	8771 (64.0)	8129 (50.6)	7827 (51.7)
Sex (*n* (%))				
Female	13,154 (57.0)	7988 (58.3)	9153 (57.0)	8719 (57.6)
Male	9902 (43.0)	5710 (41.7)	6898 (43.0)	6411 (42.4)
Race and Ethnicity (*n* (%))				
Hispanic	3024 (13.1)	1165 (8.5)	2015 (12.5)	2173 (14.4)
Non-Hispanic Black	4390 (19.1)	1802 (13.2)	3030 (18.9)	3007 (19.9)
Non-Hispanic Other	812 (3.5)	288 (2.1)	498 (3.1)	610 (4.0)
Non-Hispanic White	14,830 (64.3)	10,443 (76.2)	10,508 (65.5)	9,340 (61.7)
Asymmetry Ratio	1.1 ± 0.7	1.1 ± 0.2	1.1 ± 0.9	1.1 ± 0.3

**Table 2.** Overall Prevalence of Handgrip Strength Asymmetry.

Variables	Weighted Frequency	Weighted Prevalence (%)	95% Confidence Interval
*2006–2008 Waves*			
>10% Asymmetry	31,788,352	52.3	51.2, 53.3
>20% Asymmetry	15,006,125	24.7	23.8, 25.5
>30% Asymmetry	6,863,484	11.3	10.7, 11.9
*2010–2012 Waves*			
>10% Asymmetry	39,408,290	52.5	51.5, 53.5
>20% Asymmetry	18,121,546	24.2	23.3, 24.9
>30% Asymmetry	8,203,994	10.9	10.3, 11.5
*2014–2016 Waves*			
>10% Asymmetry	41,768,747	53.4	52.2, 54.4
>20% Asymmetry	20,333,356	26.0	25.0, 26.9
>30% Asymmetry	9,122,294	11.7	10.9, 12.3

**Table 3.** Prevalence of Handgrip Strength Asymmetry by Age Group.

Variables	Weighted Frequency	Weighted Prevalence (%)	95% Confidence Interval
**Middle-Aged**			
*2006–2008 Waves*			
>10% Asymmetry	15,685,369	50.0	48.4, 51.6
>20% Asymmetry	6,986,534	22.3	20.9, 23.6
>30% Asymmetry	3,016,437	9.6	8.6, 10.5
*2010–2012 Waves*			
>10% Asymmetry	21,658,015	50.8	49.4, 52.3
>20% Asymmetry	9,120,495	21.4	20.2, 22.6
>30% Asymmetry	3,893,630	9.1	8.3, 9.9
*2014–2016 Waves*			
>10% Asymmetry	21,602,962	52.4	50.7, 54.0
>20% Asymmetry	9,956,971	24.1	22.7, 25.5
>30% Asymmetry	4,160,854	10.1	9.1, 11.0
**Older**			
*2006–2008 Waves*			
>10% Asymmetry	16,102,983	54.6	53.5, 55.8
>20% Asymmetry	8,019,591	27.2	26.2, 28.2
>30% Asymmetry	3,847,047	13.1	12.2, 13.8
*2010–2012 Waves*			
>10% Asymmetry	17,750,275	54.6	53.4, 55.9
>20% Asymmetry	9,001,051	27.7	26.6, 28.8
>30% Asymmetry	4,310,364	13.3	12.4, 14.1
*2014–2016 Waves*			
>10% Asymmetry	20,165,785	54.3	53.0, 55.7
>20% Asymmetry	10,376,385	27.9	26.7, 29.1
>30% Asymmetry	4,961,440	13.4	12.4, 14.2

**Table 4.** Prevalence of Handgrip Strength Asymmetry by Sex.

Variables	Weighted Frequency	Weighted Prevalence (%)	95% Confidence Interval
**Females**			
*2006–2008 Waves*			
>10% Asymmetry	18,703,315	56.2	54.9, 57.5
>20% Asymmetry	9,336,260	28.1	26.9, 29.2
>30% Asymmetry	4,407,569	13.3	12.3, 14.1
*2010–2012 Waves*			
>10% Asymmetry	22,150,582	55.0	53.6, 56.2
>20% Asymmetry	10,685,138	26.5	25.3, 27.6
>30% Asymmetry	5,001,443	12.4	11.5, 13.2
*2014–2016 Waves*			
>10% Asymmetry	23,885,791	56.7	55.2, 58.0
>20% Asymmetry	12,182,138	28.9	27.6, 30.1
>30% Asymmetry	5,755,008	13.7	12.7, 14.6
**Males**			
*2006–2008 Waves*			
>10% Asymmetry	13,085,037	47.5	45.9, 49.1
>20% Asymmetry	5,669,865	20.6	19.3, 21.8
>30% Asymmetry	2,455,915	8.9	8.0, 9.7
*2010–2012 Waves*			
>10% Asymmetry	17,257,708	49.7	48.1, 51.2
>20% Asymmetry	7,436,408	21.4	20.1, 22.6
>30% Asymmetry	3,202,551	9.2	8.3, 10.0
*2014–2016 Waves*			
>10% Asymmetry	17,882,956	49.5	47.8, 51.1
>20% Asymmetry	8,151,218	22.6	21.2, 23.8
>30% Asymmetry	3,367,286	9.3	8.4, 10.2

**Table 5.** Prevalence of Handgrip Strength Asymmetry by Race and Ethnicity.

Variables	Weighted Frequency	Weighted Prevalence (%)	95% Confidence Interval
**Hispanic**			
*2006–2008 Waves*			
>10% Asymmetry	2,307,885	54.3	50.7, 57.9
>20% Asymmetry	1,093,585	25.7	22.7, 28.7
>30% Asymmetry	579,443	13.6	11.3, 15.9
*2010–2012 Waves*			
>10% Asymmetry	3,256,286	54.1	50.9, 57.3
>20% Asymmetry	1,602,089	26.6	23.8, 29.4
>30% Asymmetry	789,090	13.1	11.0, 15.2
*2014–2016 Waves*			
>10% Asymmetry	3,926,489	54.8	51.7, 57.9
>20% Asymmetry	2,003,204	27.9	25.1, 30.7
>30% Asymmetry	999,833	14.0	11.6, 16.2
**Non-Hispanic Black**			
*2006–2008 Waves*			
>10% Asymmetry	2,912,082	55.3	52.5, 58.2
>20% Asymmetry	1,546,295	29.4	26.8, 31.9
>30% Asymmetry	722,081	13.7	11.8, 15.6
*2010–2012 Waves*			
>10% Asymmetry	3,791,707	52.7	50.3, 55.0
>20% Asymmetry	1,916,715	26.6	24.5, 28.6
>30% Asymmetry	954,539	13.3	11.6, 14.8
*2014–2016 Waves*			
>10% Asymmetry	4,310,761	55.8	53.3, 58.2
>20% Asymmetry	2,243,122	29.0	26.7, 31.2
>30% Asymmetry	1,192,640	15.4	13.5, 17.3
**Non-Hispanic Other**			
*2006–2008 Waves*			
>10% Asymmetry	806,124	53.5	46.5, 60.4
>20% Asymmetry	438,082	29.0	22.6, 35.5
>30% Asymmetry	220,700	14.6	9.5, 19.7
*2010–2012 Waves*			
>10% Asymmetry	1,374,429	54.0	48.5, 59.5
>20% Asymmetry	598,602	23.5	18.7, 28.3
>30% Asymmetry	306,303	12.0	8.3, 15.7
*2014–2016 Waves*			
>10% Asymmetry	1,959,416	55.2	49.8, 60.6
>20% Asymmetry	914,638	25.8	21.1, 30.4
>30% Asymmetry	437,089	12.3	9.1, 15.5
**Non-Hispanic White**			
*2006–2008 Waves*			
>10% Asymmetry	25,762,261	51.7	50.6, 52.9
>20% Asymmetry	11,928,163	23.9	23.2, 24.9
>30% Asymmetry	5,341,260	10.7	10.0, 11.4
*2010–2012 Waves*			
>10% Asymmetry	30,985,868	52.2	51.1, 53.4
>20% Asymmetry	14,004,140	23.6	22.6, 24.5
>30% Asymmetry	6,154,062	10.4	9.7, 11.0
*2014–2016 Waves*			
>10% Asymmetry	31,572,081	52.7	51.4, 54.0
>20% Asymmetry	15,172,392	25.3	24.2, 26.4
>30% Asymmetry	6,492,732	10.8	10.0, 11.6

**Table 6.** Results for the Handgrip Strength Asymmetry Categories Trends Analyses.

Variables	>10% Asymmetry			>20% Asymmetry			>30% Asymmetry		
	Estimate	95% CI	*p*-value	Estimate	95% CI	*p*-value	Estimate	95% CI	*p*-value
Overall Model									
Intercept	0.12	0.07, 0.18	<0.001	−1.10	−1.2, −1.0	<0.001	−2.00	−2.10, −1.90	<0.001
Wave	0.01	−0.02, 0.03	0.52	0.02	−0.01, 0.05	0.11	0.01	−0.02, 0.05	0.41
Age Group Model									
Intercept	−0.03	−0.11, 0.06	0.50	−1.30	−1.40, −1.20	<0.001	−2.20	−2.30, −2.10	<0.001
Wave	0.05	0.01, 0.08	0.01	0.05	0.01, 0.09	0.02	0.03	−0.02, 0.09	0.26
Older	0.23	0.13, 0.34	<0.001	0.27	0.15, 0.39	<0.001	0.29	0.13, 0.46	<0.001
Wave*Older	−0.05	−0.10, −0.01	0.04	−0.02	−0.07, 0.03	0.45	0.01	−0.07, 0.08	0.96
Gender Model									
Intercept	0.27	0.02, 034	<0.001	−0.92	−0.99, −0.84	<0.001	−1.90	−2.00, −1.70	<0.001
Wave	−0.01	−0.05, 0.02	0.34	0.01	−0.03, 0.04	0.80	0.01	−0.04, 0.06	0.65
Male	−0.35	−0.45, −0.24	<0.001	−0.42	−0.54, −0.30	<0.001	−0.39	−0.54, −0.23	<0.001
Wave*Male	0.05	0.01, 0.10	0.02	0.05	−0.01, 0.10	0.08	0.01	−0.06, 0.09	0.72
Ethnicity Model									
Intercept	0.09	0.03, 0.15	0.002	−1.10	−1.20, −1.10	<0.001	−2.10	−2.20, −2.00	<0.001
Wave	0.01	−0.02, 0.04	0.55	0.03	−0.01, 0.06	0.12	0.01	−0.03, 0.06	0.54
Hispanic	0.19	002, 0.37	0.03	0.26	0.06, 0.45	0.01	0.44	0.17, 0.71	0.001
Non-Hispanic Black	0.14	−0.01, 0.29	0.06	0.24	0.08, 0.41	0.004	0.22	−0.01, 0.44	0.06
Non-Hispanic Other	−0.10	−0.44, 0.23	0.54	0.06	−0.32, 0.44	0.75	0.11	−0.40, 0.63	0.66
Wave*Hispanic	−0.05	−0.13, 0.03	0.20	−0.06	−0.15, 0.02	0.14	−0.12	−0.24, −0.01	0.04
Wave*Non-Hispanic Black	−0.02	−0.08, 0.55	0.56	−0.03	−0.10, 0.04	0.43	0.01	−0.09, 0.11	0.87
Wave*Non-Hispanic Other	0.05	−0.09, 0.20	0.45	−0.03	−0.20, 0.13	0.68	−0.01	−0.22, 0.21	0.95

## Data Availability

The dataset generated from (or analyzed in) the study can be found at the Health and Retirement Study website: https://hrs.isr.umich.edu/data-products.

## ABBREVIATIONS

HGS: handgrip strength
HRS: Health and Retirement Study
