# Supplementary material for: Prevalence and Trends of Handgrip Strength Asymmetry in the United States
Source: Adv Geriatr Med Res. Author manuscript; Available in PMC 2023 Jul 27. (PMC10373124; doi:10.20900/agmr20230006)
Supplement: Supplementary material [file NIHMS1914293-supplement-Supplementary_material.pdf]

Supplementary File 1. Overall Prevalence of the Separated Categories for Handgrip Strength Asymmetry.

	Weighted Frequency	Weighted Prevalence (%)	95% Confidence Interval
2006-2008 Waves			
0.0-10.0% Asymmetry	28,985,733	47.7	46.7, 48.7
10.1-20.0% Asymmetry	16,782,227	27.6	26.7, 28.5
20.1-30.0% Asymmetry	8,142,641	13.4	12.7, 14.1
>30.0% Asymmetry	6,863,484	11.3	10.7, 11.9
2010-2012 Waves			
0.0-10.0% Asymmetry	35,614,639	47.5	46.4, 48.4
10.1-20.0% Asymmetry	21,286,744	28.4	27.4, 29.2
20.1-30.0% Asymmetry	9,917,552	13.2	12.5, 13.8
>30.0% Asymmetry	8,203,994	10.9	10.3, 11.5
2014-2016 Waves			
0.0-10.0% Asymmetry	36,516,410	46.6	45.5, 47.7
10.1-20.0% Asymmetry	21,435,391	27.4	26.4, 28.3
20.1-30.0% Asymmetry	11,211,062	14.3	13.5, 15.0
>30.0% Asymmetry	9,122,294	11.7	10.9, 12.3

Supplementary File 2. Prevalence of the Separated Categories for Handgrip Strength Asymmetry by Age Group.

	Weighted Frequency	Weighted Prevalence (%)	95% Confidence Interval
**Middle-Aged**			
2006-2008 Waves			
0.0-10.0% Asymmetry	15,644,957	49.9	48.3, 51.5
10.1-20.0% Asymmetry	8,698,835	27.8	26.3, 29.2
20.1-30.0% Asymmetry	3,970,097	12.7	11.5, 13.7
>30.0% Asymmetry	3,016,437	9.6	8.6, 10.5
2010-2012 Waves			

0.0-10.0% Asymmetry	20,912,419	49.1	47.6, 50.5
10.1-20.0% Asymmetry	12,537,520	29.5	28.1, 30.7
20.1-30.0% Asymmetry	5,226,865	12.3	11.3, 13.2
>30.0% Asymmetry	3,893,630	9.1	8.3, 9.9
2014-2016 Waves			
0.0-10.0% Asymmetry	19,610,588	47.6	45.9, 49.2
10.1-20.0% Asymmetry	11,645,991	28.3	26.7, 29.7
20.1-30.0% Asymmetry	5,796,117	14.1	12.9, 15.2
>30.0% Asymmetry	4,160,854	10.1	9.1, 11.0
**Older**			
2006-2008 Waves			
0.0-10.0% Asymmetry	13,340,776	45.3	44.1, 46.4
10.1-20.0% Asymmetry	8,083,392	27.5	26.4, 28.4
20.1-30.0% Asymmetry	4,172,544	14.2	13.3, 14.9
>30.0% Asymmetry	3,847,047	13.1	12.2, 13.8
2010-2012 Waves			
0.0-10.0% Asymmetry	14,702,220	45.3	44.0, 46.5
10.1-20.0% Asymmetry	8,749,224	27.0	25.8, 28.0
20.1-30.0% Asymmetry	4,690,687	14.5	13.5, 15.3
>30.0% Asymmetry	4,310,364	13.3	12.4, 14.1
2014-2016 Waves			
0.0-10.0% Asymmetry	16,905,822	45.6	44.2, 46.9
10.1-20.0% Asymmetry	9,789,400	26.4	25.2, 27.5
20.1-30.0% Asymmetry	5,414,945	14.6	13.6, 15.5
>30.0% Asymmetry	4,961,440	13.4	12.4, 14.2

Supplementary File 3. Prevalence of the Separated Categories for Handgrip Strength Asymmetry by Sex.

	Weighted Frequency	Weighted Prevalence (%)	95% Confidence Interval
**Females**			
2006-2008 Waves			
0.0-10.0% Asymmetry	14,550,006	43.8	42.4, 45.0
10.1-20.0% Asymmetry	9,367,055	28.2	26.9, 29.3
20.1-30.0% Asymmetry	4,928,691	14.8	13.8, 15.7
>30.0% Asymmetry	4,407,569	13.3	12.3, 14.1
2010-2012 Waves			
0.0-10.0% Asymmetry	18,145,430	45.0	43.7, 46.3
10.1-20.0% Asymmetry	11,465,444	28.5	27.2, 29.6
20.1-30.0% Asymmetry	5,683,695	14.1	13.2, 15.0
>30.0% Asymmetry	5,001,443	12.4	11.5, 13.2
2014-2016 Waves			
0.0-10.0% Asymmetry	18,254,713	43.3	41.9, 44.7
10.1-20.0% Asymmetry	11,703,653	27.8	26.4, 29.0
20.1-30.0% Asymmetry	6,427,130	15.3	14.2, 16.2
>30.0% Asymmetry	5,755,008	13.7	12.7, 14.6
**Males**			
2006-2008 Waves			
0.0-10.0% Asymmetry	14,435,727	52.5	50.8, 54.0
10.1-20.0% Asymmetry	7,415,172	26.9	25.5, 28.3
20.1-30.0% Asymmetry	3,213,950	11.7	10.6, 12.6
>30.0% Asymmetry	2,455,915	8.9	8.0, 9.7
2010-2012 Waves			
0.0-10.0% Asymmetry	17,469,209	50.3	48.7, 51.8
10.1-20.0% Asymmetry	9,821,300	28.3	26.9, 29.6

20.1-30.0% Asymmetry	4,233,857	12.2	11.2, 13.1
>30.0% Asymmetry	3,202,551	9.2	8.3, 10.0
2014-2016 Waves			
0.0-10.0% Asymmetry	18,261,697	50.5	48.8, 52.1
10.1-20.0% Asymmetry	9,731,738	26.9	25.4, 28.3
20.1-30.0% Asymmetry	4,783,932	13.2	12.1, 14.3
>30.0% Asymmetry	3,367,286	9.3	8.4, 10.2

Supplementary File 4. Prevalence of the Separated Categories for Handgrip Strength Asymmetry by Race and Ethnicity.

	**Weighted Frequency**	**Weighted Prevalence (%)**	**95% Confidence Interval**
**Hispanic**			
2006-2008 Waves			
0.0-10.0% Asymmetry	1,937,579	45.6	42.0, 49.2
10.1-20.0% Asymmetry	1,214,300	28.6	25.3, 31.8
20.1-30.0% Asymmetry	514,142	12.1	9.9, 14.2
>30.0% Asymmetry	579,443	13.6	11.3, 15.9
2010-2012 Waves			
0.0-10.0% Asymmetry	2,757,147	45.8	42.6, 49.0
10.1-20.0% Asymmetry	1,654,197	27.5	24.6, 30.3
20.1-30.0% Asymmetry	812,999	13.5	11.3, 15.6
>30.0% Asymmetry	789,090	13.1	11.0, 15.2
2014-2016 Waves			
0.0-10.0% Asymmetry	3,231,754	45.1	42.0, 48.2
10.1-20.0% Asymmetry	1,923,285	26.9	24.1, 29.6
20.1-30.0% Asymmetry	1,003,371	14.0	12.0, 16.0
>30.0% Asymmetry	999,833	14.0	11.6, 16.2
**Non-Hispanic Black**			

2006-2008 Waves			
0.0-10.0% Asymmetry	2,345,550	44.6	41.7, 47.4
10.1-20.0% Asymmetry	1,365,787	26.0	23.5, 28.4
20.1-30.0% Asymmetry	824,214	15.7	13.5, 17.7
>30.0% Asymmetry	722,081	13.7	11.8, 15.6
2010-2012 Waves			
0.0-10.0% Asymmetry	3,405,716	47.3	44.9, 49.7
10.1-20.0% Asymmetry	1,874,992	26.1	23.9, 28.1
20.1-30.0% Asymmetry	962,176	13.4	11.8, 14.9
>30.0% Asymmetry	954,539	13.3	11.6, 14.8
2014-2016 Waves			
0.0-10.0% Asymmetry	3,411,969	44.2	41.7, 46.6
10.1-20.0% Asymmetry	2,067,639	26.8	24.5, 28.9
20.1-30.0% Asymmetry	1,050,482	13.6	11.9, 15.2
>30.0% Asymmetry	1,192,640	15.4	13.5, 17.3
**Non-Hispanic Other**			
2006-2008 Waves			
0.0-10.0% Asymmetry	700,397	46.5	39.5, 53.4
10.1-20.0% Asymmetry	368,042	24.4	18.4, 30.4
20.1-30.0% Asymmetry	217,382	14.4	9.4, 19.3
>30.0% Asymmetry	220,700	14.6	9.5, 19.7
2010-2012 Waves			
0.0-10.0% Asymmetry	1,168,373	45.9	40.4, 51.4
10.1-20.0% Asymmetry	775,827	30.5	25.2, 37.7
20.1-30.0% Asymmetry	292,299	11.5	7.9, 15.0
>30.0% Asymmetry	306,303	12.0	8.3, 15.7
2014-2016 Waves			

0.0-10.0% Asymmetry	1,585,547	44.7	39.3, 50.1
10.1-20.0% Asymmetry	1,044,778	29.5	24.4, 34.4
20.1-30.0% Asymmetry	477,549	13.5	9.6, 17.3
>30.0% Asymmetry	437,089	12.3	9.1, 15.5
**Non-Hispanic White**			
2006-2008 Waves			
0.0-10.0% Asymmetry	24,002,207	48.2	47.0, 49.3
10.1-20.0% Asymmetry	13,834,098	27.8	26.7, 28.8
20.1-30.0% Asymmetry	6,586,903	13.2	12.4, 14.0
>30.0% Asymmetry	5,341,260	10.7	10.0, 11.4
2010-2012 Waves			
0.0-10.0% Asymmetry	28,283,403	47.7	46.5, 48.8
10.1-20.0% Asymmetry	16,981,728	28.7	27.5, 29.7
20.1-30.0% Asymmetry	7,850,078	13.2	12.4, 14.0
>30.0% Asymmetry	6,154,062	10.4	9.7, 11.0
2014-2016 Waves			
0.0-10.0% Asymmetry	28,287,140	47.3	45.9, 48.5
10.1-20.0% Asymmetry	16,399,689	27.4	26.2, 28.5
20.1-30.0% Asymmetry	8,679,660	14.5	13.5, 15.4
>30.0% Asymmetry	6,492,732	10.8	10.0, 11.6

Supplementary File 5. Prevalence of Handgrip Strength Asymmetry in Young Old, Middle Old, and Old-Old.

	Weighted Frequency	Weighted Prevalence (%)	95% Confidence Interval
**Young Old**			
2006-2008 Waves			
>10% Asymmetry	8,390,113	52.8	51.2, 54.3
>20% Asymmetry	4,022,544	25.3	23.9, 26.6
>30% Asymmetry	1,731,413	10.8	9.9, 11.8
2010-2012 Waves			

>10% Asymmetry	9,530,344	52.8	50.9, 54.6
>20% Asymmetry	4,662,737	25.8	24.2, 27.4
>30% Asymmetry	2,087,817	11.5	10.4, 12.7
2014-2016 Waves			
>10% Asymmetry	11,707,019	53.4	51.4, 55.3
>20% Asymmetry	5,761,432	26.3	24.5, 28.0
>30% Asymmetry	2,614,004	11.9	10.6, 13.1
**Middle Old**			
2006-2008 Waves			
>10% Asymmetry	5,723,810	56.0	54.1, 58.0
>20% Asymmetry	2,893,825	28.3	26.5, 30.1
>30% Asymmetry	1,477,239	14.4	13.0, 15.8
2010-2012 Waves			
>10% Asymmetry	6,081,716	56.6	54.7, 58.2
>20% Asymmetry	3,197,040	29.7	28.0, 31.5
>30% Asymmetry	1,634,189	15.2	13.8, 16.5
2014-2016 Waves			
>10% Asymmetry	5,993,824	54.9	53.0, 56.9
>20% Asymmetry	3,214,009	29.4	27.7, 31.2
>30% Asymmetry	1,586,636	14.5	13.1, 15.9
**Old-Old**			
2006-2008 Waves			
>10% Asymmetry	1,989,060	59.4	55.9, 62.8
>20% Asymmetry	1,103,222	32.9	29.6, 36.2
>30% Asymmetry	638,395	19.0	16.3, 21.8
2010-2012 Waves			
>10% Asymmetry	2,138,215	58.3	54.8, 61.7
>20% Asymmetry	1,141,274	31.1	27.9, 34.3
>30% Asymmetry	588,358	16.0	13.5, 18.5
2014-2016 Waves			
>10% Asymmetry	2,464,942	57.7	54.4, 61.1
>20% Asymmetry	1,400,944	32.8	29.6, 36.0
>30% Asymmetry	760,800	17.8	15.2, 20.4
